# Maximum kinetic energy considerations in proton stereotactic radiosurgery

**DOI:** 10.1120/jacmp.v12i3.3533

**Published:** 2011-04-12

**Authors:** Evan R. Sengbusch, Thomas R. Mackie

**Affiliations:** ^1^ Department of Medical Physics University of Wisconsin School of Medicine and Public Health Madison WI 53705; ^2^ Departments of Medical Physics Human Oncology, Biomedical Engineering, and Engineering Physics University of Wisconsin School of Medicine and Public Health Madison WI 53705 USA

**Keywords:** proton therapy, stereotactic radiosurgery, arc therapy, radiation therapy

## Abstract

The purpose of this study was to determine the maximum proton kinetic energy required to treat a given percentage of patients eligible for stereotactic radiosurgery (SRS) with coplanar arc‐based proton therapy, contingent upon the number and location of gantry angles used. Treatment plans from 100 consecutive patients treated with SRS at the University of Wisconsin Carbone Cancer Center between June of 2007 and March of 2010 were analyzed. For each target volume within each patient, in‐house software was used to place proton pencil beam spots over the distal surface of the target volume from 51 equally‐spaced gantry angles of up to 360°. For each beam spot, the radiological path length from the surface of the patient to the distal boundary of the target was then calculated along a ray from the gantry location to the location of the beam spot. This data was used to generate a maximum proton energy requirement for each patient as a function of the arc length that would be spanned by the gantry angles used in a given treatment. If only a single treatment angle is required, 100% of the patients included in the study could be treated by a proton beam with a maximum kinetic energy of 118 MeV. As the length of the treatment arc is increased to 90°, 180°, 270°, and 360°, the maximum energy requirement increases to 127, 145, 156, and 179 MeV, respectively. A very high percentage of SRS patients could be treated at relatively low proton energies if the gantry angles used in the treatment plan do not span a large treatment arc. Maximum proton kinetic energy requirements increase linearly with size of the treatment arc.

PACS numbers: 87.55.‐x, 87.53.Ly, 87.53.Bn

## I. INTRODUCTION

The dosimetric characteristics of protons give them several theoretical advantages over photons for use in radiation therapy, such as their finite range and Bragg peak. Such considerations have been widely reported on in the literature. However, proton therapy is still only available to a relatively small percentage of radiation therapy patients. As of February 2011, there were only nine operating proton facilities in the United States, compared to over 2000 radiation therapy treatment centers. The primary reason that proton therapy has such limited availability is that the equipment used in proton therapy is much larger and more expensive than the equipment used in traditional photon radiation therapy. Several novel accelerator designs have been proposed for use in proton therapy, as will be discussed in more detail below. In all cases, the maximum energy required of the accelerator is the critical factor in the size and cost of the treatment system. This is due to the fact that the costs of the accelerator and magnet systems increase as beam energy increases.

In 2009, a paper was published that quantified the maximum proton kinetic energy requirements for a set of 100 radiation therapy patients that were randomly selected to be representative of the total pool of patients eligible for radiation therapy.^(1)^ That work specifically excluded patients that underwent cranial stereotactic radiosurgery (SRS). This work is meant to be an extension of that study and focuses on patients treated with SRS for cranial diseases. Over 600,000 patients to date have been treated by dedicated photon SRS treatment machines, such as the Accuray CyberKnife and the Elekta Leksell Gamma Knife, and over 500 of these systems are now treating patients across the globe. Furthermore, a comparable number of patients receive SRS treatments from standard linacs. Even though proton radiotherapy was used by Lars Leksell for the first stereotactic patients, the use of protons for SRS in clinical practice is today extremely limited. Several properties of protons, such as their finite range^(2)^ and reduced integral dose relative to photons^(^ ^3^ ^,^ ^4^ ^)^, make them particularly attractive for use in SRS. Furthermore, those treatment volumes that are close to the skull can most benefit from proton radiotherapy because the entrance path, and therefore the integral dose to normal brain tissue, is reduced. Tumors closer to the surface need less energy, but could perhaps benefit from shallow angle arc delivery to spread out the entrance dose to a wider area.

The increased cost of protons relative to photons^(5)^ is a result of the increased level of technology required to create a clinically useful proton beam. There are currently several new accelerator technologies that are being developed for use in proton radiation therapy, including small cryogenic cyclotrons,^(6)^ plasma wakefield accelerators,^(^ ^7^ ^,^ ^8^ ^)^ and the dielectric wall accelerator (DWA).^(9)^ For the majority of these new technologies, as well as technologies that are currently in use (i.e., cyclotrons and synchrotrons), the maximum proton kinetic energy that is required of the accelerator plays the critical role in the total size and cost of the treatment system. Decreasing the maximum energy requirements of a proton system could reduce the physical space and cost requirements, and potentially increase the availability of this treatment modality to patients. This study focuses on the energy necessary for the design of an arc‐based proton system, which would use a coplanar delivery geometry typically used for rotational therapy.

It has been shown that approximately 90% to 95% of patients eligible for proton therapy can be treated with coplanar arc‐based delivery with proton energies at or below 200 MeV.^(1)^ However, not all treatment sites are ideally suited for treatment with protons. For example, although a relatively large percentage of patients who receive proton radiation therapy are treated for prostate cancer, the advantage of protons over photons for this disease site has been called into question.^(10)^ Some of the characteristics of a disease site that would be ideally suited for proton therapy include: shallow target location, nearby or adjacent critical structures with strict dose limits, and few or no tissue heterogeneities that run parallel to the treatment beam. Most or all of these characteristics are present in the majority of patients with cranial diseases and in most SRS patients. Furthermore, since cranial treatments generally require lower proton energies than some other disease sites, it is natural to conceive of a proton machine designed specifically to accommodate brain diseases.

In this study, the maximum proton kinetic energy that would be required for a treatment system dedicated to brain SRS patients is estimated. This value depends strongly on the gantry angles from which the treatment beam is incident. While only coplanar directions are used in this study, it is likely that entrance directions that minimize the path length to the tumor are optimal. Thus, the estimates presented here represent a conservative estimate of the maximum energy needed. Further discussion of non‐coplanar beam directions is presented in the following sections below. This study presents a comprehensive analysis to determine the distribution of required energies as a function of the length of the coplanar treatment arc.

## II. MATERIALS AND METHODS

One hundred patients treated consecutively from June 2007 through March 2010 with photon SRS at the University of Wisconsin Carbone Cancer Center were included in this study. All patients had diseases of the brain and were treated on a Varian linear accelerator with a tertiary collimator system. A summary of the diseases included is shown in Table 1. For each patient, CT data and ROI contours were exported from the Pinnacle (Philips Medical Systems, Fitchburg, WI) treatment planning system and imported to in‐house software for analysis. The CT information was converted to density and for each patient, voxel sizes of 0.0703 cm×0.0703 cm×0.125 cm were used, where the last dimension is the slice thickness. For each target ROI in each patient, maximum proton energies were calculated for 51 coplanar gantry angles equally spaced over 360°. A discretization of 51 beam angles was chosen because it coincides with that used by the TomoTherapy rotational treatment planning software (TomoTherapy Inc., Madison, WI), with which there exists extensive clinical experience, and because that number of beam angles was on the high end of what was computationally feasible for this study.

**Table 1:** Maximum proton energy requirements in rotational stereotactic radiosurgery.

			*With Arc Length of:*		
*Disease*	Patients in Study	Single Angle	90°	180°	*360°*
Metastases	51	106	117	145	179
Trigeminal Neuralgia	22	105	124	133	151
Vestibular Schwannoma	5	111	124	137	163
Meningioma	7	103	111	133	177
Arteriovenous Malformation	3	118	127	142	172
Astrocytoma	5	98	109	140	169
Glioblastoma Multiforme	4	110	120	136	177
Other	3	100	126	129	151
Total	100	118	127	145	179

The table above shows the maximum proton energy required for treatment with stereotactic radiosurgery of all patients in this study, separated by disease, as a function of the length of the treatment arc. In all cases, the gantry angle or treatment arc was selected to minimize the proton energy requirement for each patient. Proton energy values are accurate to within 2%, as discussed in the text.

**Figure 1:**
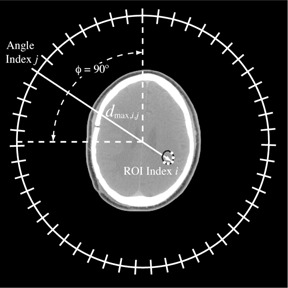
Sample patient with a brain metastasis. The tick marks around the outer circle show the gantry angles from which proton beam energies were calculated for each target volume. The target volume with index *i* is shown in black. Sample distal edge beam spots are shown as white dots along the boundary of the target (not to scale). The maximum path length $d^{\text{max},i,j}$ is shown originating from the gantry angle indexed by *j*, where in this example, ϕ was taken to be 90°.

The procedure for calculating the maximum proton energy for a single gantry angle and a single target ROI is as follows. Voxels were classified based on their density, and were considered to be air (below $0.005\;{g/\text{cm}}^{2}$), adipose tissue (between $0.005\;{g/\text{cm}}^{2}$ and $1.100\;{g/\text{cm}}^{2}$), muscle (between $1.100\;{g/\text{cm}}^{2}$ and $1.500\;{g/\text{cm}}^{2}$), or bone (greater than $1.500\;{g/\text{cm}}^{2}$). These cutoffs are based on clinical values and have a negligible effect on the results of this study. Each voxel was then scaled by the ratio of the mass stopping power of its tissue type to that of water. The scaling factors that were used were the average value taken over the energy range 50 MeV to 250 MeV, as reported by NIST.^(11)^ The values used for air, adipose tissue, muscle, and bone were 1.029, 0.884, 0.930, and 0.989, respectively. Within this energy range, the mass stopping power ratios do not vary by more than 0.5% for any of these tissue types. The result was an effective water‐equivalent density grid that accounted for differences in tissue types and stopping powers. It is important to note that there are inherent uncertainties when converting CT numbers to density and stopping power values. A similar study using a less accurate range calculation algorithm showed that the error in the proton kinetic energy requirements due to inaccuracies in stopping power conversion factors was less than 2%.^(1)^ Other studies have set an upper limit on stopping power uncertainties of 1.8%,^(12)^ which corresponds to an error in proton energy of less than 1%. While a 2% energy uncertainty would be critical for the treatment plan of a single patient, it is not nearly as crucial in the context of this study, which seeks merely to give general guidance on energy requirements for a dedicated coplanar arc‐based proton system. A grid mask of beam spots was then placed to cover the entire target volume. The beam spots were separated laterally and in depth by 0.235 cm. This value was chosen because it corresponds to 40% of the full width at half maximum (FWHM) of a Gaussian proton beamlet with a standard deviation of 0.25 cm at the surface of the patient, and it has been shown that such a spot spacing method is more than adequate to create uniform dose distributions within a target using spot scanning proton therapy.^(13)^ A ray tracing algorithm^(14)^ was used to select those spots that fell closest to the distal surface of the target, as recognized by transitions from target to nontarget voxels. Along each of these rays, the effective radiological path length from the beam origin to the distal spot was calculated by summing the product of the distance traveled through each voxel and the effective water‐equivalent density value of that voxel. A maximum was then taken over the effective radiological path lengths for all beam spots to give $d_{\text{max},i,j}$, where *i* is the index for the target ROI and *j* is the index for the gantry angle for the given patient, as shown in Fig. 1.

Each maximum path length $d_{\text{max},i,j}$ was then converted to a proton energy $E_{\text{max},i,j}$ by finding the energy of a proton pencil beam (with parameters given above and incident on water) that had a Bragg peak depth that was closest to $d_{\text{max},i,j}$. Beamlets were calculated between 50 and 250 MeV using Monte Carlo simulation with MCNPX, which has been benchmarked with experiment for proton therapy and other physics applications,^(^ ^15^ ^–^ ^23^ ^)^ with an energy separation of 1 MeV. This method essentially places the Bragg peak on the distal bounding of the target, which gives an estimate of the maximum energy required. For each of the 100 patients, $E_{\text{max},i,j}$ was calculated for all *i* target ROIs and all 51 gantry angles. It was then possible to determine the maximum proton energy required to treat each patient as a function of the arc length ϕ through which treatment was required. A search was done for the minimum path length necessary to deliver the required arc segment anywhere within the 360° set of possible coplanar beam directions. If we let *k* be the index for the set of all groups of consecutive treatment angles spanning ϕ degrees and we let *n* serve as the patient index, then this maximum proton kinetic energy value for a given treatment arc length ϕ can be represented mathematically as $E_{n}(\phi )=\underset{i}{\max}\left[\underset{k(j)}{\min}(E_{\max,i,j})\right]$. These values were calculated for several different values of ϕ, namely 1°, 90°, 180°, 270°, and 360°, and the results are shown in the following section. An arc length of 1° represents a single beam direction.

An example may help to clarify the procedure. Suppose ϕ is fixed to be 90°. Consider a single ROI in a single patient. First, the maximum proton energy required to reach the distal edge of the target was calculated for the arc spanning 0° to 90°. Next, the arc was shifted by 7.059° to get an arc spanning the angles 7.059° to 97.059°, and the maximum proton energy was calculated for that arc. This procedure was repeated until a maximum energy value was obtained for all 51 possible arcs spanning 90°. The minimum was then taken over this set of 51 maximum energy values to give the proton energy requirement for that ROI in that patient. This procedure was repeated with different values of ϕ for all ROIs and all patients to produce the results presented in the following section. It is important to note that this method of using arcs for this analysis does not necessarily require arc treatment for these results to be applicable. Included within the arc treatment results are static gantry angle results. The arc method simply ensures that all possible coplanar treatment angles (discretized into 51 beam angles for tractability) were considered, and that an upper bound was set on the proton energy requirements. Note that since all smaller arcs are inherently included in any larger arcs, the maximum energy requirement is necessarily an increasing function of ϕ.

## III. RESULTS & DISCUSSION

**Figure 2:**
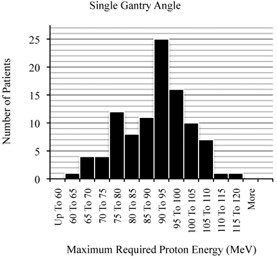
Single gantry angle delivery proton energy requirements. The histogram shows the number of patients in the study requiring a given maximum proton energy for treatment with proton stereotactic radiosurgery (bin size 5 MeV). Delivery is from a single gantry angle, and that angle was chosen to minimize the proton energy requirement for each patient. Proton energy values are accurate to within 2% percent, as discussed in the text.

**Figure 3:**
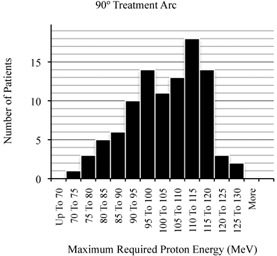
90° arc delivery proton energy requirements. The histogram shows the number of patients in the study requiring a given maximum proton energy for treatment with proton stereotactic radiosurgery (bin size 5 MeV). Delivery is over a continuous arc of 90°, and the arc was chosen to minimize the proton energy requirement for each patient. Proton energy values are accurate to within 2%, as discussed in the text.

**Figure 4:**
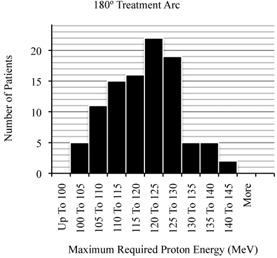
180° arc delivery proton energy requirements. The histogram shows the number of patients in the study requiring a given maximum proton energy for treatment with proton stereotactic radiosurgery (bin size 5 MeV). Delivery is over a continuous arc of 180°, and the arc was chosen to minimize the proton energy requirement for each patient. Proton energy values are accurate to within 2%, as discussed in the text.

**Figure 5:**
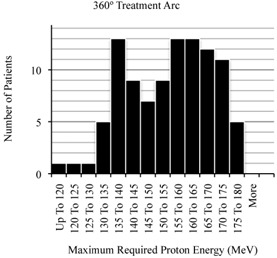
360° arc delivery proton energy requirements. The histogram shows the number of patients in the study requiring a given maximum proton energy for treatment with proton stereotactic radiosurgery (bin size 5 MeV). Delivery is over a continuous arc of 360°. Proton energy values are accurate to within 2%, as discussed in the text.

The results of the calculations described above are shown in Figs. 2–5 and Table 1. When interpreting these results, it is important to keep in mind that the energy estimates apply to the required beam energy immediately prior to entering the patient. For the DWA‐based scanning beam system that has been proposed, this is very close to the energy required of the accelerator since the beam will only pass through monitor chambers after exiting the accelerator and before entering the patient. However, for other systems in which the beam passes through additional material (e.g., scattering foils, compensators), the energy requirements must be increased to account for the loss in energy attributed to interactions in these materials. Since each system has a different amount of energy reduction prior to patient entry, the results presented here represent energy requirements immediately prior to patient entry and can easily be modified based on the specific physical properties of the system in question. Furthermore, a margin for range uncertainties was not included in this study. Although in photon‐based SRS it is uncommon to add margins, it is common in current clinical proton practice to include margins as great as 1 cm. Adding such margins on the distal side of the target could increase the energy estimates recorded in this study by as much as 3.5%, and this clinical concern should be kept in mind when interpreting the results presented here.

It is clear from these results that the energy requirements for proton SRS in brain patients are considerably less than for the general population of all patients eligible for proton radiotherapy. For example, the energy requirement to treat nearly 100% of eligible patients with proton therapy over a 180° treatment arc is approximately 240 MeV.^(1)^ However, in this study, all 100 SRS patients could be treated over 180° with a maximum proton energy of only 145 MeV. Furthermore, if the length of the treatment arc is decreased to 90°, which is sufficient for most small tumors, 100% of the patients could be treated with a maximum energy of 130 MeV, and 95% could be treated with an energy below 120 MeV. It is possible to reduce this energy requirement even further if treatment from only a single gantry angle is considered. Depending on the normal tissues present in the entry path, it may be possible to treat from only a single gantry angle. If this were the case, 100% of patients in this study could be treated below 120 MeV, and 98% could be treated below 110 MeV. These results suggest that a proton system dedicated to SRS could have reduced energy requirements. This could lead to reductions in the size and price of such a system, as discussed in greater detail below.

**Figure 6:**
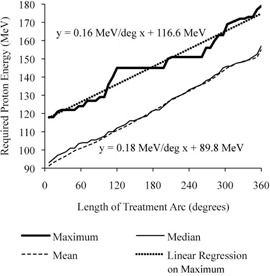
Required proton treatment energy as a function of treatment arc length. Shown are the maximum, median, and mean of the maximum proton energy required to treat each patient with proton stereotactic radiosurgery, taken over all 100 patients. Linear regressions were run on the maximum and median datasets, and the equations generated by the regression are shown on the graph. The regression curve for the maximum energies is shown as the dashed line, but was omitted for the median energies (for the sake of easier readability). Proton energy values are accurate to within 2%, as discussed in the text.

It is clear from the discussion above that as the required length of the treatment arc increases, so does the maximum proton energy requirement. Figure 6 shows the maximum, median, and mean of the maximum proton energy requirements as a function of the required treatment arc length, taken over all 100 patients. As can be seen from this figure, the mean and median for this dataset are very similar. Furthermore, they vary in a surprisingly linear fashion with the length of the treatment arc. The linear regressions are meant only to characterize the general correlation between the maximum energy and the treatment arc length.

As can be seen in Fig. 1, shallow tumors should not be treated with arc paths through 360° because there is no need to treat through the ipsilateral side of the brain and deliver unnecessary integral dose. The integral dose for a central tumor would be nearly invariant with arc length, but there are neurological reasons to avoid proton dose from one side of the brain and, hence, the use of 360° arc delivery would be unwarranted. A previous study has suggested that 360° arc delivery may not be superior to 180° arc delivery.^(24)^ Thus, it is hard to conceive of a need for a cranial system with energies above 145 MeV. Energy requirements for 360° arc treatments were included for completeness only. It is unlikely that such a treatment would be used regularly in clinical practice.

There is no mention in the analysis above of the avoidance of critical structures, which is clearly an extremely important consideration in stereotactic radiosurgery. However, this study does not seek to specify desirable beam angles for individual treatment plans. Certainly, some angles included in the analyses presented would never be used in an actual treatment. By considering all possible treatment angles (discretized into 51 beam directions for tractability), this study sets an upper bound on the energy requirements – the stated purpose of the study. Removing certain angles from the calculations based on critical structure constraints would have no effect on the results.

This study included only coplanar beam angles. In the current practice of photon stereotactic radiosurgery, non‐coplanar beam angles are used regularly (e.g., vertex fields). Considering such angles would likely change the results presented here. This study was meant to provide justification for a maximum energy selection in a coplanar arc‐based SRS proton unit. The coplanar design was selected to minimize mechanical complications in the unit. It is anticipated that the dosimetric properties of protons may eliminate the need for using non‐coplanar beam angles and the additional hardware complications that they introduce. Such considerations are beyond the scope of this paper, but are certainly an area for further investigations. Nevertheless, noncoplanar arcs chosen to minimize integral dose would require even less energy, and this study thus represents a conservative estimate for the energy required for non‐coplanar proton SRS.

A detailed analysis of the exact cost implications of increased energy for a proton system is beyond the scope of this paper because the total impact will vary with the accelerator technology under consideration. For example, in a cyclotron‐based proton center, increased energy would lead to larger magnet systems, which represent a substantial portion of the cost of a cyclotron. Nonetheless, it is worthwhile to consider one specific example to get a sense of the magnitude of the cost implications. This paper was written with the DWA‐based system that is under development in mind, and it is this system that will be used as an example. The DWA has a fixed energy gradient per unit length, and the cost of the accelerator scales linearly with its length. Thus, the cost of the accelerator scales linearly with the required energy. An increase in the required energy from 125 MeV to 250 MeV would double the cost of the accelerator. Of course, other system components whose cost is not affected by the required proton energy must be considered, such as the building, control system, imaging system, couch, and planning system. However, at least for the DWA example, the accelerator itself represents the majority of the cost of the entire proton facility (as much as 80%). If the accelerator is mounted on a gantry, the gantry can represent a large portion of the total facility cost. For a gantry‐based system, the accelerator may represent a smaller fraction of the total facility cost, perhaps closer to 50%; doubling the required energy would increase the cost of the total treatment facility by at least 50% to 80%. This increase considers only the accelerator, and does not take into account additional cost increases associated with a larger treatment room, higher magnetic field requirements for scanning magnets, and increased shielding requirements (for more details regarding the impact on shielding see Ref. 1). These concerns would not have as great an impact as that of the cost of the accelerator itself, but they would serve to make the impact that the required proton energy has on the overall price of the system even more substantial.

## IV. CONCLUSIONS

The largest factor limiting patient access to proton radiation therapy is the cost of the accelerator systems that create clinically useful proton beams. For nearly all systems either in use or proposed for use in proton radiation therapy, the maximum energy that must be achieved plays a major role in the size and cost of the accelerator and magnet systems used to transport the beam. Furthermore, certain disease sites receive much greater benefit from protons relative to photons than others. Specifically, cranial diseases have been shown to be well suited for proton therapy. This study attempted to quantify the maximum energy that would need to be reached by a proton accelerator to treat the majority of patients with brain lesions using coplanar arc‐based proton stereotactic radiosurgery. By using a randomized patient population of 100 photon SRS patients, systematic inclusion biases were eliminated. It was shown that a proton system dedicated to cranial SRS cases could be conceived with a much lower maximum attainable energy than conventional proton systems. It is possible that this reduction in energy could lead to substantial reductions in the size and cost of such a dedicated SRS system relative to other proton therapy systems.

## ACKNOWLEDGMENTS

This work was supported by the Department of Defense (DoD) through the National Defense Science & Engineering Graduate Fellowship (NDSEG) Program and by a National Science Foundation Graduate Fellowship. This work utilized the National Science Foundation and University of Wisconsin‐funded Grid Laboratory of Wisconsin (GLOW) computer cluster (NSF award number 0320708). Support from an NIH Training Grant T32 CA09206 is acknowledged.
